# Synthesis and Characterization of Novel Unsymmetrical and Symmetrical 3-Halo- or 3-Methoxy-substituted 2-Dibenzoylamino-1,4-naphthoquinone Derivatives

**DOI:** 10.3390/molecules18021973

**Published:** 2013-02-04

**Authors:** Yakini Brandy, Nailah Brandy, Emmanuel Akinboye, Malik Lewis, Claudia Mouamba, Seshat Mack, Ray J. Butcher, Alan J. Anderson, Oladapo Bakare

**Affiliations:** 1 Department of Chemistry, Howard University, Washington, DC 20059, USA; 2 Department of Natural Sciences, Bowie State University, Bowie, MD 20715, USA

**Keywords:** 1,4-naphthoquinone, imide synthesis, sodium hydride-promoted bis-acylation, unsymmetrical imide

## Abstract

Symmetrical and unsymmetrical 3-halo- or 3-methoxy- substituted 2-dibenzoylamino-1,4-naphthoquinone analogs were synthesized with an average yield of 45% via sodium hydride promoted bis-acylation of 2-amino-3-chloro-1,4-naphthoquinone, 2-amino-3-bromo-1,4-naphthoquinone and 2-amino-3-methoxy-1,4-naphthoquinone.

## 1. Introduction

The quinone moiety is an important part of many biologically active natural products and their synthetic analogs [1,2,3,4,5,6,7,8,9,10,11,12,13,14]. The quinonoid anti-cancer drugs such as mitomycin C, daunorubicin,doxorubicin and mitoxantrone have been used in the treatment of various types of cancers, including solid tumors, for many years. We have been involved in the synthesis and biological evaluation of some quinonoid compounds [15,16,17,18,19,20,21], and previously we developed 2-chloro-3-(*N*-succinimidyl)-1,4-naphthoquinone and some of its analogs as MEK1 specific inhibitors of the Ras-MAPK pathway [15]. In a subsequent report, we demonstrated the anti-carcinogenic activities of some of these imido-substituted 2-chloro-1,4-naphthoquinone derivatives on androgen-dependent, LNCaP, and androgen-independent, PC3 and DU145, human prostate cancer cell lines [16]. In continuation of our work on the development of novel imido-substituted 1,4-naphthoquinones for studies in our prostate and breast cancer drug development program, we have designed some novel bis-arylimido-substituted 1,4-naphthoquinones. The sodium hydride promoted bis-acylation of 2-amino-3-halo-1,4-naphthoquinone and 2-amino-3-methoxy-1,4-naphthoquinone to furnish some novel 3-halo- and 3-methoxy-substituted 2-dibenzoylamino-1,4-naphthoquinone analogs is therefore reported here.

## 2. Results and Discussion

**Scheme 1:**
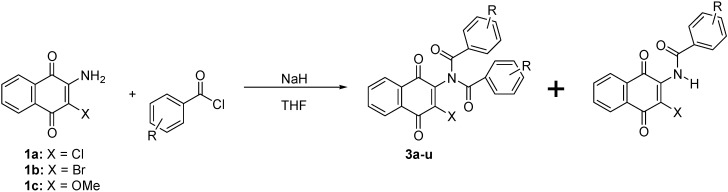
Synthesis of symmetrical 3-halo- and 3-methoxy-substituted 2-dibenzoylamino-1,4-naphthoquinone analogs.

In our ongoing studies on the chemical modification of naphthoquinones to give biologically useful compounds, we previously reported the synthesis of some cyclic and acyclic imido-2-chloro-1,4-naphthoquinones obtained by reacting 2-amino-3-chloro-1,4-naphthoquinone with acid chlorides at elevated temperatures in the absence of a base or catalyst. Similar reaction conditions did not produce the corresponding bis-arylimido-1,4-naphthoquinone analogs in any appreciable yield. Hence, we explored a base promoted bis-acylation of the starting 2-amino-3-chloro-1,4-naphthoquinone. Various organic and inorganic bases were initially employed (including triethylamine, pyridine, DMAP and Na_2_CO_3_) at room temperature and at elevated temperatures. These conditions did not afford the target imido compounds, rather, the starting 2-amino-3-chloro-1,4-naphthquinone and the amide derivatives were mostly recovered. However, the reaction of 2-amino-3-chloro-1,4-naphthoquinone (**1a)** with sodium hydride in dry THF under an inert atmosphere, followed by a nucleophilic acyl substitution reaction of the resulting nucleophile on an appropriate acid chloride furnished imido-substituted derivatives in addition to the amide analogs, as shown in Scheme 1.

Similar reaction on 2-amino-3-bromo-1,4-naphthquinone (**1b**) and 2-amino-3-methoxy-1,4-naphthoquinone (**1c**) also furnished the corresponding imido-substituted derivatives, showing the procedure proceeded equally well in the presence of either electron withdrawing or electron donating group at position 3. The target imido-substituted analogs **3a**–**u** were obtained with an average yield of 45% after purification by column chromatography on silica gel and/or recrystallization from appropriate solvents.

**Figure 1:**
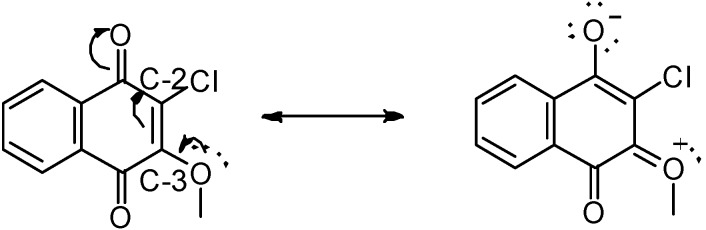
Delocalization of electrons from the methoxy group on C-3 reduces the electrophilicity of C2.

**Scheme 2:**
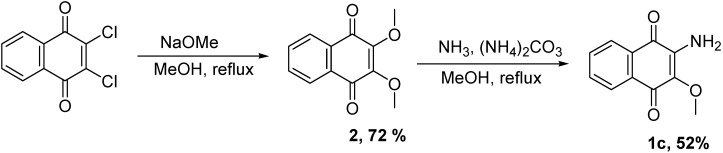
Synthesis of 2-amino-3-methoxy-1,4-naphthoquinone.

**Figure 2:**
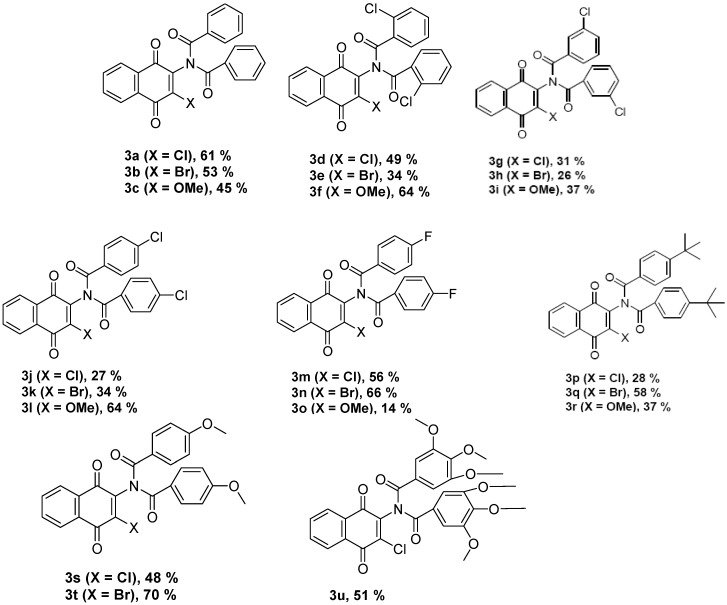
Structures of 3-halo- or 3-methoxy-substituted 2-dibenzoyl-amino-1,4-naphthoquinone analogs.

The 2-amino-3-halo-1,4-naphthoquinones **1a**,**b** were prepared from the 2,3-dichloro-1,4-naphtho-quinone as previously described for **1a** [15]. An initial attempt to obtain **1c** from 2-chloro-3-methoxy-1,4-naphthoquinone furnished the amino-chloro analog **1a** instead. This suggested substitution of the methoxy group rather than the chloro group, which is most likely due to lower electrophilicity of C2 as a result of electron delocalization from the methoxy group at C3 as shown in Figure 1. Consequently, we first synthesized 2,3-dimethoxy-1,4-naphthoquinone (**2**) from 2,3-dichloro-1,4-naphthoquinone and sodium methoxide in 72% yield as shown in Scheme 2 [22]. Subsequent reaction of **2** with ammonia in the presence of ammonium carbonate furnished 2-amino-3-methoxy-1,4-naphthoquinone (**1c**) in 52% yield (Scheme 2). The structures of all the symmetrical imido-substituted 1,4-naphthoquinones **3a**–**u** synthesized in this study are shown in Figure 2.

**Scheme 3:**
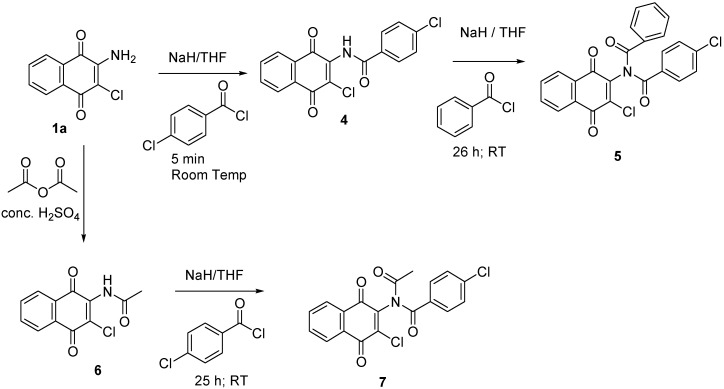
Synthesis of unsymmetrical 2-*N*-benzoyl-*N*-(4-chlorobenzoyl)amino-3-chloro-1,4-naphthoquinone (**5**) and 2-*N*-acetyl-*N*-(4-chlorobenzoyl)amino-3-chloro-1,4-naphthoquinone (**7**) analogs.

Two unsymmetrical analogs **5** and **7** were also synthesized as shown in Scheme 3 by first forming the amide derivatives **4** and **6**. The amide derivative **4** was made by heating 2-amino-3-chloro-1,4-naphthoquinone in *p*-chlorobenzoyl chloride; or by stirring 2-amino-3-chloro-1,4-naphthoquinone, sodium hydride and *p*-chlorobenzoyl chloride (1:3:1.5 mole ratio) in THF for 5 min at room temperature. Amide **6** was synthesized in excess acetic anhydride at room temperature with a catalytic amount of conc. sulfuric acid [23]. Subsequent reaction of the isolated pure amides, **4** and **6**, with NaH followed by addition of benzoyl chloride (1:2:2 mole ratio) or *p*-chlorobenzoyl chloride (1:1.5:1.3 mole ratio) furnished the unsymmetrical analogs **5** and **7** in 43% and 41 % yield, respectively. 

**Figure 3:**
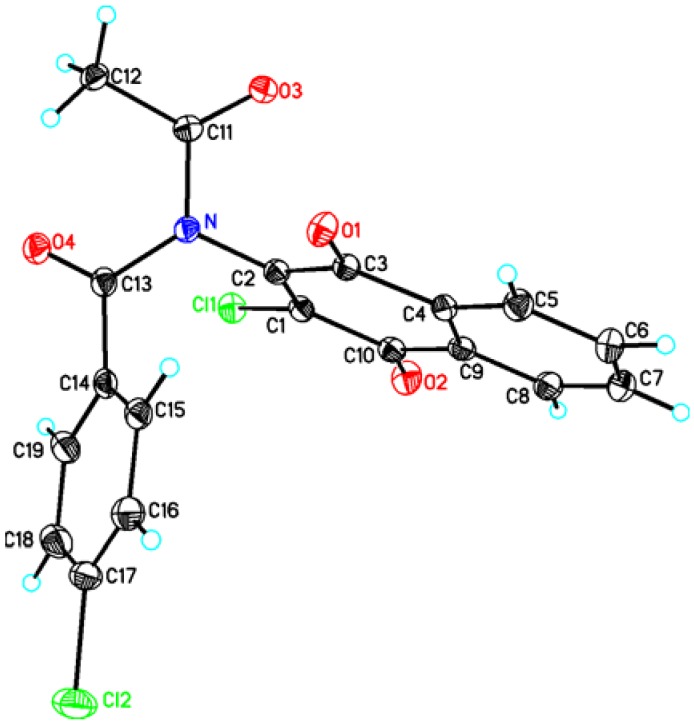
Molecular structure of compound **7**.

As shown in Figure 3, the crystal structure of compound **7** shows that the imide group is almost perpendicular to the plane of the naphthoquinone ring and the two imide carbonyls are *anti* to each other.

## 3. Experimental

### 3.1. General

Infrared spectra were recorded on a Perkin Elmer PR 100 Spectrometer equipped with an Attenuated Total Reflectance (ATR) window. ^1^H- and ^13^C-NMRs were performed on the Bruker Avance 400 MHZ spectrometer. Compounds were analyzed in deuterated chloroform (CDCl_3_), deuterated dimethyl sulfoxide (DMSO-*d_6_*). Mass spectra were obtained on a Jeol AccuTOF-CS ESI TOF, LTQ Orbitrap XL tandem Mass Spectrometer and Agilent Technologies 6224 TOF LC MS. Unless where specified, all materials were obtained from commercial sources and used without further purification.

*2-Amino-3-methoxy-1,4-naphthoquinone* (**1c**). Ammonia gas was bubbled continuously into a mixture of 2,3-dimethoxy-1,4-naphthoquinone (**2**, 0.305 mg, 1.40 mmol) and (NH_4_)_2_CO_3_ (439 mg, 4.57 mmol) in anhydrous MeOH (70 mL). The reaction mixture was refluxed at 50 °C for 2 h. Additional (NH_4_)_2_CO_3_ (418 mg, 4.35 mmol) was added to the cooled solution and the mixture was again refluxed for 1 h. Nitrogen was bubbled through the cooled solution for 10 mins. The solution was concentrated in vacuo, ice-cold water (20 mL) added to the resulting mixture, and the precipitate formed was isolated by filtration under suction. This solid was air dried to obtain a red solid (226 mg) which was re-crystallized from ethyl acetate / hexane (50:50) to obtain red solid (148 mg, 52%). Mp: 138–140 °C. IR (cm^−1^) 3425, 3313, 3246, 2948, 1670, 1638, 1607, 1579, 1564, 1352, 1221. ^1^H-NMR (CDCl_3_) 4.00 (s, 3H), 5.08 (s, 2H), 7.54–7.58 (dt, 1H, *J* = 1.28, 7.48 Hz), 7.63–7.67 (dt, 1H, *J* = 1.44, 7.56 Hz), 7.93–7.96 (dd, 1H, *J* = 1.32, 7.60 Hz), 8.00–8.03 (dd, 1H, *J* = 1.00, 7.64 Hz). ^13^C-NMR (CDCl_3_) 59.92, 125.31, 125.55, 129.74, 131.70, 131.83, 133.88, 137.06, 138.51, 177.95, 181.57. ESI HRMS *m/z* 204.0650 ([M + H]^+^ calcd 204.0661).

*2,3-Dimethoxy-1,4-naphthoquinone* (**2**). A mixture of 2,3-dichloro-1,4-naphthoquinone (4.960 g, 0.022 mol) and sodium methoxide (3.582 g, 0.066 mol) in anhydrous MeOH (100 mL) was refluxed for 5 h. A second portion of sodium methoxide (2.365 g, 0.044 mol) was added and the mixture refluxed for another hour. The reaction mixture was concentrated under vacuum, filtered and the residue repeatedly washed with cold water to obtain a yellow solid (3.463 g, 72%). Mp: 113–115 °C. IR (cm^−1^) 3014, 2957, 1673, 1651, 1590, 1320, 1268, 1211.66, 1162, 1043, 1015. ^1^H-NMR (CDCl_3_) 4.11 (s, 6H), 7.67–7.72 (m, 2H), 8.03–8.08 (m, 2H). ^13^C-NMR (CDCl_3_) 61.39, 126.21, 130.81, 133.70, 147.54, 181.93. ESI HRMS *m/z* 241.0470 ([M+Na]^+^ calcd 241.0477).

### 3.2. General Experimental for the Imido-Substituted Derivatives ***3a–u***

2-Amino-3-chloro-1,4-naphthoquinone, 2-amino-3-bromo-1,4-naphthoquinone or 2-amino-3-methoxy-1,4-naphthoquinone was dissolved in freshly distilled THF (15 mL). NaH was added and the mixture was stirred at room temperature for 15 min. The appropriate acid chloride was added, drop wise, and the mixture was stirred for 24 hours under argon (mole ratio of substrate:NaH:acid chloride = 1:2.3:2.3). THF was evaporated under vacuum and the mixture was washed with ice-water (10 g ice and 10 mL water). The ice-water mixture was extracted with CH_2_Cl_2_ (30 mL, 20 mL consecutively). The combined organic phase was washed with water (3 × 20 mL), saturated NaCl solution (3 × 20 mL), and then dried over anhydrous MgSO_4_. The crude material was purified via triturating in hot ethanol, recrystallization in ethyl acetate and/or via column chromatography.

#### Characterization Data for Each Isolated Imido-Substituted Compounds **3a–u**

*2-Dibenzoylamino-3-chloro-1,4-naphthoquinone* (**3a**). Yellow solid. (61%). Mp: 294–297 °C. IR (cm^−1^) 3328, 1698, 1672, 1618, 1590, 1573. ^1^H-NMR (DMSO-*d_6_*) 7.35–7.39 (t, 4H, *J* = 7.5 Hz), 7.46–7.50 (tt, 2H, *J* = 1.2, 7.5 Hz), 7.65–7.67 (m, 4H), 7.83–7.90 (m, 2H), 7.94–7.96 (dd, 1H, *J* = 1.7, 7.4 Hz), 8.04–8.06 (dd, 1H, *J* = 1.3, 8.2 Hz). ^13^C-NMR (CDCl_3_) 127.61, 127.64, 128.59, 129.11, 130.69, 131.37, 132.90, 134.45, 134.68, 134.73, 142.32, 144.33, 171.59, 177.29, 178.59. ESI HRMS *m/z* 438.0513 ([M+Na]^+^ calcd 438.0509).

*2-Dibenzoylamino-3-bromo-1,4-naphthoquinone* (**3b**). Yellow solid. (53%). Mp: 234–238 °C. IR (cm^−1^) 3087, 1693, 1671, 1588, 1570. ^1^H-NMR (CDCl_3_) 7.30–7.34 (t, 4H, *J* = 7.40 Hz), 7.41–7.45 (tt, 2H, *J* = 1.21, 7.49 Hz), 7.77–7.81 (m, 6H), 8.10–8.12 (m, 1H), 8.20–8.22 (m, 1H). ^13^C-NMR (CDCl_3_) 127.72, 127.96, 128.58, 129.22, 130.65, 131.22, 132.89, 134.58, 134.68, 134.70, 138.75, 147.47, 171.55, 177.48, 178.30. ESI HRMS *m/z* 482.0005 ([M+Na]^+^ calcd 482.0004).

*2-Dibenzoylamino-3-methoxy-1,4-naphthoquinone* (**3c**). Yellow solid. (45%) Mp: 262–266 °C. IR (cm^−1^) 3012, 2953, 1675, 1651, 1605, 1578, 1270, 1249, 1218. ^1^H-NMR (CDCl_3_) 4.14 (s, 3H), 7.31–7.34 (dt, 4H, *J* = 1.60, 7.28 Hz), 7.41–7.45 (tt, 2H, *J* = 1.28, 7.44 Hz), 7.73–7.76 (m, 6H), 8.04–8.09 (m, 2H). ^13^C-NMR (CDCl**_3_**) 181.22, 180.43, 172.63, 154.71, 134.89, 134.55, 134.02, 132.61, 132.56, 131.67, 131.48, 130.86, 130.26, 129.04, 128.58, 128.54, 126.91, 126.73, 61.65. ESI HRMS *m/z* 434.0998 ([M+Na]^+^ calcd 434.1004).

*2-Bis-(2-chlorobenzoyl)amino-3-chloro-1,4-naphthoquinone* (**3d**). Yellow solid. (49%). Mp: 217–218 °C. IR (cm^−1^) 1720, 1681, 1618, 1588. ^1^H-NMR (CDCl_3_) 7.13–7.16 (d, 2H, *J* = 7.91 Hz), 7.21–7.25 (dt, 2H, *J* = 1.68, 7.51 Hz), 7.27–7.31 (dt, 2H, *J* = 1.30, 7.57), 7.79–7.85 (m, 2H), 7.87–7.89 (d, 2H, *J* = 4.75 Hz), 8.15–8.20 (m, 1H), 8.22–8.26 (m, 1H). ^13^C-NMR (CDCl_3_) 126.76, 126.93, 127.60, 127.80, 129.69, 130.54, 130.85, 131.37, 132.36, 132.52, 132.56, 132.75, 134.14, 134.75, 134.91, 142.65, 144.43, 177.13, 178.22. ESI HRMS *m/z* 505.9741 ([M+Na]^+^ calcd 505.9730).

*2-Bis-(2-chlorobenzoyl)amino-3-bromo-1,4-naphthoquinone* (**3e**). Yellow crystal (34%). Mp: 232–234 °C. IR (cm^−1^) 1728, 1688, 1671, 1588, 1468. ^1^H-NMR (CDCl_3_) 7.12–7.14 (d, 2H, *J* = 7.88 Hz), 7.19–7.23 (dd, 2H, *J* = 1.64, 7.56 Hz), 7.27–7.31 (dt, 2H, *J* = 1.28, 7.60 Hz), 7.80–7.85 (m, 2H), 7.93–7.94 (d, 2H, 5.2 Hz), 8.16–8.20 (m, 1H), 8.23–8.27 (m, 1H). ^13^C-NMR (CDCl_3_) 126.65, 127.66, 128.08, 129.63, 130.49, 130.81, 131.23, 132.25, 134.68, 134.80, 140.78, 145.81, 167.57, 177.26, 177.81. ESI HRMS *m/z* 549.9240 ([M+Na]^+^ calcd 549.9224).

*2-Bis-(2-chlorobenzoyl)amino-3-methoxy-1,4-naphthoquinone* (**3f**). Yellow solid. (64%) Mp: 195–200 °C. IR (cm^−1^) 3090, 2959, 1718, 1673, 1658, 1620, 1590, 1263, 1218, 1234, ^1^H-NMR (CDCl_3_) 4.30 (s, 3H), 7.19–7.28 (m, 6H), 7.73–7.80 (m, 4H), 8.07–8.12 (m, 2H). ^13^C-NMR (CDCl_3_) 180.88, 179.74, 168.21, 155.31, 134.30, 133.61, 132.08, 131.67, 131.10, 130.57, 130.16, 129.32, 128.67, 126.46, 126.27, 115.41, 103.74, 102.58, 61.47. ESI HRMS *m/z* 502.0224 ([M+Na]^+^ calcd 502.0225).

*2-Bis-(3-chlorobenzoyl)amino-3-chloro-1,4-naphthoquinone* (**3g**). Yellow solid. (31%). Mp: 258–260 °C. IR (cm^−1^) 3075, 1713, 1698, 1672, 1591, 1571. ^1^H-NMR (CDCl_3_) 7.27–7.31 (t, 2H, *J* = 7.85 Hz), 7.42–7.45 (ddd, 2H, *J* = 1.03, 2.09, 8.07 Hz), 7.60–7.63 (td, 2H, *J* = 1.07, 7.68 Hz), 7.70–7.71 (t, 2H, *J* = 1.82), 7.80–7.85 (m, 2H), 8.11–8.14 (m, 1H), 8.21–8.23 (m, 1H). ^13^C-NMR (CDCl_3_) 127.02, 127.93, 128.03, 129.34, 130.21, 130.75, 131.55, 133.29, 135.14, 135.21, 136.06, 143.00, 143.84, 170.20, 177.25, 178.66. ESI HRMS *m/z* 505.9741 ([M+Na]^+^ calcd 505.9730).

*2-Bis-(3-chlorobenzoyl)amino-3-bromo-1,4-naphthoquinone* (**3h**). Yellow solid (26%) Mp: 204–206 °C. IR (cm^−1^) 3074, 1698, 1671, 1589, 1566. ^1^H-NMR (CDCl_3_) 7.27–7.30 (t, 2H, *J* = 7.92 Hz), 7.41–7.44 (ddd, 2H, *J* = 1.04, 2.08, 8.04 Hz), 7.62–7.65 (td, 2H, *J* = 1.12, 7.72 Hz), 7.72–7.73 (t, 2H, *J* = 1.84 Hz), 7.78–7.85 (m, 2H), 8.10–8.14 (m, 1H), 8.20–8.24 (m, 1H). ^13^C-NMR (CDCl_3_) 127, 128.09, 128.40, 129.49, 130.25, 130.78, 131.49, 133.32, 135.15, 135.18, 135.24, 136.25, 139.56, 147.05, 170.21, 177.50, 178.43. ESI HRMS *m/z* 549.9236 ([M+Na]^+^ calcd 549.9224).

*2-Bis-(3-chlorobenzoyl)amino-3-methoxy-1,4-naphthoquinone* (**3i**). Yellow solid. (37%) Mp: 182–184 °C. IR (cm^−1^) 3107, 2960, 1707, 1689, 1670, 1657, 1604, 1572, 1283, 1266, 1239, 1218. ^1^H-NMR (CDCl_3_) 4.19 (s, 3H), 7.26–7.30 (t, 2H, *J* = 7.984 Hz), 7.40–7.43 (ddd, 2H, *J* = 1.04, 2.08, 8.04 Hz), 7.58–7.61 (td, 2H, *J* = 1.16, 7.72 Hz), 7.68–7.69 (t, 2H, *J* = 1.84 Hz), 7.73–7.77 (m, 2H), 8.05–8.09 (m, 2H). ^13^C-NMR (CDCl_3_) 180.66, 179.85, 170.63, 154.37, 135.97, 134.53, 134.35, 133.83, 132.37, 131.06, 130.29, 130.21, 129.58, 128.60, 126.58, 126.51, 126.32, 61.53. ESI HRMS *m/z* 502.0219 ([M+Na]^+^ calcd 502.0225).

*2-Bis-(4-chlorobenzoyl)amino-3-chloro-1,4-naphthoquinone* (**3j**). Yellow solid. (27%). Mp: 212–213 °C. IR (cm^−1^) 1737, 1714, 1696, 1673, 1589, 1571. ^1^H-NMR (CDCl_3_) 7.33–7.36 (td, 4H, *J* = 2.34, 8.54 Hz), 7.68–7.72 (td, 4H, *J* = 2.40, 8.47 Hz), 7.79–7.85 (m, 2H), 8 09–8.11 (dd, 1H, *J* = 2.50, 6.56 Hz), 8.20–8.22 (dd, 1H, *J* = 2.40, 6.66 Hz). ^13^C-NMR (CDCl_3_) 127.72, 127.80, 129.18, 130.34, 130.50, 131.32, 132.52, 134.92, 134.95, 139.67, 142.46, 143.87, 170.40, 177.05, 178.59. ESI MS *m/z* 505.975 ([M+Na]^+^ calcd 505.973).

*2-Bis-(4-chlorobenzoyl)amino-3-bromo-1,4-naphthoquinone* (**3k**). Yellow solid. (34%). Mp: 266–269 °C. IR (cm^−1^) 3096, 1703, 1661, 1586. ^1^H-NMR (CDCl_3_) 7.33–7.36 (td, 4H, *J* = 2.16, 8.47 Hz), 7.70–7.73 (td, 4H, *J* = 2.37, 8.53 Hz), 7.79–7.82 (m, 2H), 8.08–8.11 (m, 1H), 8.20–8.22(m, 1H). ^13^C-NMR (CDCl_3_) 127.80, 128.09, 129.13, 130.41, 130.46, 131.17, 132.66, 134.84, 134.90, 138.94, 139.61, 146.98, 170.32, 177.21, 178.28. ESI MS *m/z* 549.923 ([M+Na]^+^ calcd 549.9224).

*2-Bis-(4-chlorobenzoyl)amino-3-methoxy-1,4-naphthoquinone* (**3l**). Yellow solid. (64%) Mp: 170–172 °C. IR (cm^−1^) 3100, 2958, 1722, 1687, 1668, 1687, 1652, 1589, 1575, 1274. ^1^H-NMR (CDCl_3_) 4.15 (s, 3H), 7.32–7.35 (td, 4H, *J* = 2.36, 8.64 Hz), 7.66–7.70 (td, 4H, *J* = 2.36, 8.68 Hz), 7.74–7.76 (m, 2H), 8.05–8.08 (m, 2H). ^13^C-NMR (CDCl_3_) 181.11, 180.45, 171.50, 154.73, 139.32, 134.80, 134.32, 133.05, 131.52, 131.08, 130.75, 130.30, 129.19, 127.04, 126.97, 61.93. ESI HRMS *m/z* 502.0223 ([M+Na]^+^ calcd 502.0225).

*2-Bis-(4-fluorobenzoyl)amino-3-chloro-1,4-naphthoquinone* (**3m**). Yellow solid. (56%). Mp: 284–286 °C. IR (cm^−1^) 3074, 1719, 1689, 1672, 1591. ^1^H-NMR (CDCl_3_) 7.00–7.06 (tt, 4H, *J* = 2.85, 8.71 Hz), 7.75–7.85 (m, 6H), 8.10–8.12 (m, 1H), 8.20–8.22 (m, 1H). ^13^C-NMR (CDCl_3_) 116.08 (d, ^2^J_F,C_ = 22.2 Hz, C-C-F), 116.41, 126.65, 126.96, 127.67, 127.77, 130.53, 130.57, 130.58, 131.35, 131.66 (d, ^3^J_F,C_ = 9.4 Hz, C-C-C-F), 132.75, 134.88, 142.42, 144.13, 165.41(d, ^1^J_F,C_ = 255.99 Hz, C-F), 170.33, 177.13, 178.63. ESI MS *m/z* 474.034 ([M+Na]^+^ calcd 474.032).

*2-Bis-(4-fluorobenzoyl)amino-3-bromo-1,4-naphthoquinone* (**3n**). Orange-yellow solid (66%). Mp: 170–172 °C. IR (cm^−1^) 1719, 1670, 1596, 1505. ^1^H-NMR (CDCl_3_) 7.03–7.07 (tt, 4H, *J* = 2.04, 8.68 Hz), 7.80–7.84 (m, 6H), 8.11–8.14 (m, 1H), 8.23–8.25 (m, 1H).^13^C-NMR (CDCl_3_) 115.68 (d, ^2^J_F,C_ = 22.2 Hz, C-C-F), 127.4, 127.71, 130.18, 130.30, 130.33, 130.85, 131.39 (d, ^3^J_F,C_ = 9.3 Hz, C-C-C-F), 131.44, 134.46, 134.50, 138.52, 146.90, 165.03 (d, ^1^J_F,C_ = 255.92 Hz, C-F), 169.91, 176.96, 177.97. ESI HRMS *m/z* 517.9785 ([M+Na]^+^ calcd 517.9815).

*2-Bis-(4-fluorobenzoyl)amino-3-methoxy-1,4-naphthoquinone* (**3o**). Yellow solid. (14%) Mp: 177–179 °C. IR (cm^−1^) 3073, 3015, 2957, 1708, 1672, 1652, 1614, 1600, 1589, 1269. ^1^H-NMR (CDCl_3_) 8.09–8.04 (m, 2H), 7.78–7.74 (m, 6H), 7.03–7.01 (t, 4H, *J* = 8.56 Hz), 4.15 (s, 3H). ^13^C-NMR (CDCl**_3_**) 61.63, 115.82 (d, ^2^J_F,C_ = 22.5 Hz, C-C-F), 126.70, 126.78, 130.60, 130.79, 130.82, 131.22, 131.30 (d, ^3^J_F,C_ = 8.9 Hz, C-C-C-F), 131.31, 134.03, 134.52, 146.62, 154.51, 165.18 (d, ^1^J_F,C_ = 255.2 Hz, C-F), 166.45, 171.20, 180.29, 180.95. ESI HRMS *m/z* 470.0807 ([M + Na]^+^ calcd 470.0816).

*2-Bis-(4-tert-butylbenzoyl)amino-3-chloro-1,4-naphthoquinone* (**3p**). Yellow solid.(28%). Mp: 257–260 °C. IR (cm^−1^) 2965, 2901, 2868, 1701, 1673, 1603, 1592, 1572. ^1^H-NMR (CDCl_3_) 1.25 (s, 18H), 7.30–7.32 (td, 4H, *J* = 1.81, 8.53 Hz), 7.67–7.69(td, 4H, *J* = 1.86, 8.57 Hz), 7.78–7.81 (m, 2H), 8.09–8.13 (m, 1H), 8.22–8.18 (m, 1H). ^13^C-NMR (CDCl_3_) 30.96, 35.06, 125.51, 127.60, 127.65, 129.14, 130.82, 131.45, 131.71, 134.62, 134.68, 142.09, 144.58, 156.57, 171.66, 177.51, 178.67. ESI HRMS *m/z* 550.1740 ([M+Na]^+^ calcd 550.1761).

*2-Bis-(4-tert-butylbenzoyl)amino-3-bromo-1,4-naphthoquinone* (**3q**). Yellow solid. (58%) Mp: 244–250 °C. IR (cm^−1^) 2965, 1699, 1674, 1604, 1270, 1186. ^1^H-NMR (CDCl_3_) 1.24 (s, 18H), 7.29–7.31 (d, 4H, *J* = 8.40 Hz), 7.68–7.70 (d, 4H, *J* = 8.4 Hz), 7.77–7.80 (m, 2H), 8.09–8.11 (m, 1H), 8.19–8.21 (m, 1H). ^13^C-NMR (CDCl_3_) 30.75, 34.85, 125.26, 127.52, 127.69, 129.03, 130.58, 131.10,m131.65, 134.36, 134.40, 138.26, 147.50, 156.30, 171.39, 177.46, 178.15. ESI HRMS *m/z* 1165.3845 ([dimer+Na]^+^ calcd 1165.2614).

*2-Bis-(4-tert-butylbenzoyl)amino-3-methoxy-1,4-naphthoquinone* (**3r**). Yellow solid. (37%) Mp: 222–224 °C. IR (cm^−1^) 2964, 2905, 2868, 1693, 1674, 1662, 1605, 1271, 1220, 1187. ^1^H-NMR (CDCl_3_) 1.25 (s, 18H), 4.12 (s, 3H), 7.30–7.32 (d, 4H, *J* = 8.44 Hz), 7.65–7.67 (d, 4H, *J* = 8.40 Hz), 7.71–7.75 (m, 2H), 8.04–8.10 (m, 2H). ^13^C-NMR (CDCl_3_) 30.97, 31.02, 34.98, 61.46, 125.35, 125.83, 126.53, 126.81, 128.87, 130.49, 130.87, 131.40, 131.96, 132.04, 133.81, 134.33, 154.64, 156.03, 172.52, 180.39, 181.22. ESI HRMS *m/z* 524.2449 ([M+Na]^+^ calcd 524.2437).

*2-Bis-(4-methoxybenzoyl)amino-3-chloro-1,4-naphthoquinone* (**3s**). Yellow solid. (48%). Mp: 283–287 °C. IR (cm^−1^) 3019, 1698, 1668, 1599, 1574, 1508. ^1^H-NMR (CDCl_3_).3.81 (s, 6H), 6.80–6.84 (td, 4H, *J* = 2.86, 8.95 Hz), 7.73–7.82 (m, 6H), 8.09–8.11 (m, 1H), 8.19–8.21 (m, 1H). ^13^C-NMR (CDCl_3_) 55.48, 113.98, 114.21, 126.76, 127.57, 127.64, 130.38, 130.79, 131.41, 131.48, 134.61, 134.66, 141.69, 144.95, 163.34, 171.03, 177.48, 178.77. ESI MS *m/z* 498.074 ([M+Na]^+^ calcd 498.072).

*2-Bis-(4-methoxybenzoyl)amino-3-bromo-1,4-naphthoquinone* (**3t**). Yellow solid. (70%). Mp: 205–210 °C. IR (cm^−1^) 2839, 1694, 1669, 1598, 1574, 1509, 1167. ^1^H-NMR (CDCl_3_). 3.80 (s, 6H), 6.80–6.82 (d, 4H, *J* = 8.48 Hz), 7.75–7.78 (m, 6H), 8.07–8.09 (m, 1H), 8.18–8.20 (m, 1H). ^13^C-NMR (CDCl_3_) 55.43, 113.89, 126.86, 127.67, 127.82, 130.70, 131.21, 131.52, 134.52, 134.54, 138.01, 148.01, 163.25, 170.92, 177.60, 178.40. ESI MS *m/z* 557.9965 ([M+K]^+^ calcd 557.9955).

*2-Bis-(3,4,5-trimethoxybenzoyl)amino-3-chloro-1,4-naphthoquinone* (**3u**). Orange crystals (51%). Mp: 171–172 °C. IR (cm^−1^) 3015, 2939, 2838, 1704, 1675, 1586, 1122. ^1^H-NMR (CDCl_3_) 3.81 (s, 12H), 3.84 (s, 6H), 7.02 (s, 4H), 7.81–7.84 (m, 2H), 8.12–8.14 (m, 1H), 8.22–8.24 (m, 1H). ^13^C-NMR (CDCl_3_) 56.27, 56.35, 60.93, 127.62, 127.77, 129.31, 130.69, 131.39, 134.86, 142.06, 142.27, 144.45, 153.01, 171.08, 177.27, 178.84. ESI MS *m/z* 618.118 ([M+Na]^+^ calcd 618.114).

*2-N-(4-Chlorobenzoyl))-amino-3-chloro-1,4-naphthoquinone* (**4**). 2-Amino-3-chloro-1,4-naphthoquinone (302 mg, 1.45 mmol) was dissolved in freshly distilled THF (15 mL). NaH (104 mg, 4.33 mmol) was added to the solution and the mixture was stirred at room temperature for 30 mins. *p*-Chlorobenzoyl chloride (0.27 mL, 2.14 mmol) was added and the mixture was stirred for 5 mins. THF was removed under vacuum and the mixture was extracted (see general experimental for work up). The crude was recrystallized in EtOH (20 mL) then triturated in ethyl acetate to obtain the **4** in 40% yield. IR (cm^−1^) 3330.36, 1693.64, 1659.97, 1609.37. ^1^H-NMR (CDCl_3_) 7.50–7.54 (td, *J* = 2.66, 8.45 Hz, 2H), 7.76–7.84 (m, 2H), 7.91–7.94 (td, *J* = 2.54, 8.58 Hz, 2H), 8.13–8.16 (dd, *J* = 1.86, 7.15 Hz, 1H), 8.22–8.24 (dd, *J* = 1.54, 7.46 Hz, 1H), 8.30 (s, 1H). ^13^C-NMR (DSMO-*d_6_*) 127.21, 127.30, 129.14, 129.91, 130.73, 131.57, 131.92, 132.68, 135.12, 137.16, 137.96, 142.11, 164.73, 177.93, 178.91. ESI HRMS *m/z* 346.0017 ([M+H]^+^ calcd 346.0038).

*2-(N-Benzoyl-N-(4-chlorobenzoyl))-amino-3-chloro-1,4-naphthoquinone* (**5**). Compound **4** (101 mg, 0.292 mmol) was dissolved in THF (15 mL). NaH (14 mg, 0.583 mmol) was added followed by benzoyl chloride (0.07 mL, 0.604 mmol). The resulting mixture was stirred at room temperature for 26 hours. THF was removed under vacuum and the mixture was extracted (see general experiment for work up). The crude was purified by column chromatography with silica gel in CH_2_Cl_2_ to obtain a yellow solid in 43% yield. IR (cm^−1^) 3093, 1705, 1693, 1673, 1587, 1571, 1521. ^1^H-NMR (CDCl_3_) 7.30–7.33 (td, 2H, *J* = 1.89, 6.72 Hz), 7.33–7.37 (t, *J* = 7.98 Hz, 2H), 7.45–7.47 (t, 1H, *J* = 7.46 Hz), 7.69–7.72 (td, 2H, *J* = 1.96, 8.60 Hz), 7.73–7.76 (td, 2H, *J* = 1.41, 8.49 Hz), 7.79–7.82 (m, 1H), 8.10–8.13 (m, 1H), 8.20–8.22 (m, 1H). ^13^C-NMR (CDCl_3_) 127.68, 127.73, 128.76, 129.00, 129.06, 130.63, 131.37, 133.21, 134.82, 124.84, 171.34, 177.19, 178.60. ESI MS *m/z* 472.0097 ([M+Na]^+^ calcd 472.0119).

*2-N-acteylamino-3-chloro-1,4-naphthoquinone* (**6**). 2-Amino-3-chloro-1,4-naphthoquinone (305 mg, 1.47 mmol) was stirred for 10 mins at room temperature in acetic anhydride (9 mL, 95.39 mmol). Conc. sulfuric acid (1 drop) was added and the reaction mixture was stirred to obtain a yellow precipitate in 64% yield. IR (cm^−1^) 3308.59, 1711.06, 1607.38, 1586.41, 1475.28, 1198.03. ^1^H-NMR (CDCl_3_) 2.32 (s, 3H), 7.68 (s, 1H), 7.76–7.83 (m, 2H), 8.12–8.15 (ddd, 1H, *J* = 1.88, 2.20, 7.20 Hz), 8.20–8.22 (ddd, 1H, *J* = 1.44, 2.68, 7.08 Hz). ^13^C-NMR (CDCl_3_) 24.18, 127.09, 127.57, 130.26, 131.58, 134.16, 134.83. MALDI MS *m/z* 273.1471 ([M+Na]^+^ calcd 272.0090).

*2-(N-acetyl-N-(4-chlorobenzoyl))-amino-3-chloro-1,4-naphthoquinone* (**7**). Compound **6** (300 mg, 1.2 mmol) was dissolved in THF (26 mL). NaH (75.6 mg, 1.88 mmol) was added to the solution and the mixture was stirred at room temperature for 15 min. 4-Chlorobenzoyl chloride (0.2 mL, 1.56 mmol) was added and the mixture was stirred at room temperature for 25 hrs. THF was removed under vacuum and the mixture was extracted (see general experiment for work up). The crude was purified by triturating in 50% ether/hexane mixture, followed by recrystallization from hexane (1.2 mg of crude/mL of hexane) to obtain a yellow solid in 41% yield. Mp: 134–136 °C. IR (cm^−1^) 1673.43, 1702.76, 1591.38, 1254.29, 1197.10. ^1^H-NMR (CDCl_3_) 2.56 (s, 3H), 7.31–7.35 (td, 2H, *J* = 2.32, 8.72 Hz), 7.59–7.62 (td, 2H, *J* = 2.36, 8.52 Hz), 7.79–7.82 (m, 2H), 8.11–8.16 (m, 2H). ^13^C-NMR (CDCl_3_) 25.33, 127.67, 129.02, 129.54, 130.54, 131.16, 132.67, 134.76, 134.84, 139.39, 143.32, 143.42, 169.66, 171.94, 176.85, 178.48. ESI MS *m/z* 410.0009 ([M+Na]^+^ calcd 409.9963). Structural information of compound **7** has been deposited with the CCDC as CCDC894544. This data is available free of charge from www.ccdc.com.ac.uk/conts/rtrieving.html (or from the Cambridge Crystallographic Data Centre, 12 Union Road, Cambridge CB2 IEZ, UK).

## 4. Conclusions

In conclusion, we have synthesized a series of novel symmetrical and unsymmetrical 3-halo- or 3-methoxy- substituted 2-imido-1,4-naphthoquinone analogs (with an average yield of 45%) via sodium hydride promoted bis-acylation of 3-halo-/3-methoxy-substituted 2-amino-1,4-naphthoquinone derivatives. These imido-naphthoquinone analogs were charaterized by electrospray ionization mass spectrometric studies, infrared, ^1^H-NMR and ^13^C-NMR spectroscopic techniques. X-ray crystallographic studies of an unsymmeterical analog showed the imide group is oriented almost perpendicular to the plane of the naphthoquinone ring and the two imide carbonyls are *anti* to each other.
